# Improving the efficiency of estrus synchronization in cows

**DOI:** 10.5455/javar.2024.k753

**Published:** 2024-03-31

**Authors:** Mardan Julanov, Kumis Jumatayeva, Kanat Koibagarov, Orynbay Tagayev, Yerbulat Baitlessov, Nursulu Julanova

**Affiliations:** 1 Kazakh National Agrarian Research University, Almaty, Kazakhstan; 2 Zhangir Khan University, Uralsk, Kazakhstan; 3 Faculty of Veterinary Medicine–Skopje, Ss. Cyril and Methodius University in Skopje, Skopje, Republic of North Macedonia

**Keywords:** Fertility, cows, ASD-2f, electrostimulation, estradiol-17β, estrus

## Abstract

**Objective:**

This study aimed to determine the effects of biologically active substances and electrical stimulation of the uterus in cows on the effectiveness of estrus synchronization.

**Materials and Methods:**

Ninety *(n* = 90) Kazakh white-headed cows were synchronized with two injections of gonadotropin-releasing hormone on days 0 and 9 and prostaglandin F2α on day 7. The cows were divided into six groups and, during the protocol, treated with biologically active substances (Tetramag, Selevetum, antiseptic-stimulator Dorogov 2 fraction, groups 2, 3, and 4). Cows in groups 5 and 6 were treated with the same substances but additionally had electrical stimulation of the uterus, while cows in group 1 were left untreated and served as a control.

**Results:**

The results have shown that on Day 0, no differences were observed in E2 concentrations between the groups. However, on the 10th day, a significant disparity was noted in the E2 level among cows in group 6 compared to groups 2, 3, 4, and the control group. Conversely, no significant differences were observed between groups 5 and 6. Likewise, the fertility rate in cows from group 6 was significantly higher compared to groups 2, 3, 4, and the control group, with no significant differences between groups 5 and 6.

**Conclusion:**

It can be concluded that the utilization of electrical stimulation of the uterus and the inclusion of certain biological substances during the estrus synchronization protocol demonstrate a positive effect on the reproductive performance of beef cattle in Kazakhstan.

## Introduction

Reproduction is one of the main directions of livestock development. In beef cattle breeding, one of the most common methods of reproduction management is estrus synchronization. It allows obtaining seasonal calving at the desired period of the year [1]. Nevertheless, the effectiveness of the synchronization largely depends on the properly selected and implemented protocols. Therefore, when applying innovative scientific ideas, additional adjustments to cow reproductive system stimulation methods are relevant [2].

The efficiency of estrus synchronization is influenced by many factors, including the physiological state of cows, lactation, body condition scoring, cyclicity, and the stage of the estrous cycle [3]. Many scientists have been focused on synchronization protocols that are based on three main schemes. The first is through the use of prostaglandin F2α (PGF2α), which causes lysis of the corpus luteum (CL) [4–6]. The second is the alternate use of PGF2α and gonadotropin-releasing hormone (GnRH) [7–9], while the third is the use of progestogens that mimic the function of the CL [10].

It has been reported that PGF2α contributed to the regression of the CL, allowing the growth of follicles and increasing blood E2 levels, estrus behavior, and ovulation [11]. Their combined use with GnRH created favorable conditions for the neuro-humoral regulation of sexual processes and ovulation [12,13]. Along with the latter, there is evidence that the combination of phertadinum and surfagon (PGF2α and GnRH, respectively) increases the cows’ fertility by up to 78.3% [14]. In addition, when the estrous cycle was synchronized with Dinolytic (a synthetic analog of PGF2α) and Fertagyl (a GnRH analog), an increase in cow fertility of up to 80% was observed [15].

The use of non-specific stimulants such as antiseptic-stimulator Dorogov 2 fraction (ASD-2f), Еlеоvit, and others had a positive effect on reducing the insemination index (1.8) [16]. In addition, a tissue preparation (ASD-2f) in combination with Tetramag significantly stimulated involutional changes in the genitalia and reduced the days open [17].

Furthermore, there is evidence that electrical nerve stimulation in combination with the use of homeopathic remedies restores the tone of the uterus, which shortens the days open [18]. Stimulation of the sexual function of females by vasectomized males has a positive effect on the neuroendocrine function and fertility of animals [19]. In general, other authors have also reported the positive impact of some physiological stimulation methods [20,21].

We hypothesized that biologically active substances and electrical stimulation of the uterus might influence the effectiveness of estrus synchronization in beef cows. Therefore, this study aimed to determine the effect of biologically active substances and electrical genital stimulation on the effectiveness of estrus synchronization.

## Materials and Methods

### Ethical approval

The study was approved by the Institutional Supervisory Board of the Kazakh National Agrarian Research University of Almaty (protocol code 104-D of October 28, 2020). Scientific and educational work using experimental animals is regulated by the Law of the Republic of Kazakhstan “On Veterinary Medicine” dated July 10, 2002 No. 339, the Rules for conducting preclinical studies, biomedical experiments, and clinical trials in the Republic of Kazakhstan, approved by the Order of the Minister of Health of the Republic of Kazakhstan dated July 25, 2007 No. 442.

### Experimental animals

The study was carried out at the Agricultural Production Cooperative “Azamat-2” in the Beskaragai district of the East Kazakhstan region on a Kazakh white-headed beef breed.

These animals were free from infectious and parasitic diseases, and were kept on a pasture 24 hours per day. However, they are driven to a special enclosure during synchronization protocols and fertilization implementation.

The study included a total of 117 cows. Nevertheless, obese, lean (based on the BSC [22]), lame, and mastitic cows, as well as cows with reproductive disorders, were excluded from the study, resulting in 90 cows *(n =* 90) suitable for synchronization. The cows were divided into six groups, with 15 cows in each group. Cows of all groups were kept in the same conditions and grazed on pasture. They were not supplied with additional feed. The pasture vegetation consisted of perennial grasses: creeping wheatgrass (*Elytrigia repens*), wheatgrass (Agropýron), goldenrod (Solidágo), clover (Trifólium), and licorice (*Glycyrrhiza aspera*).

### Synchronization schemes and study groups

**Figure 1.**
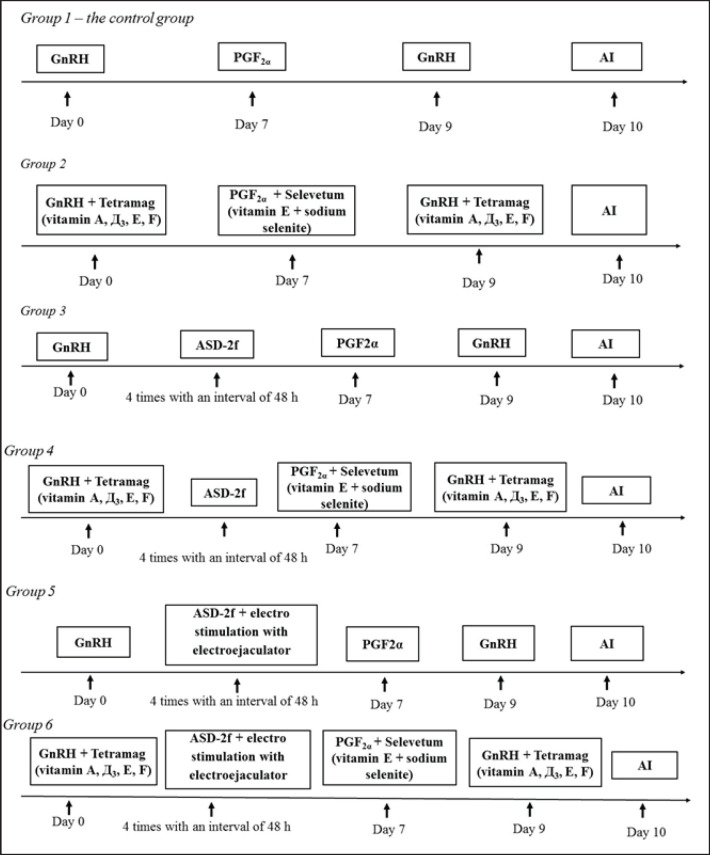
Synchronization protocols of the group.

During gynecological examinations, a trans-rectal ultrasound machine (Mindray Z5, China) and AlphaVision equipment (Imv Technologies, France) were used for vaginal examinations. The cows were synchronized using two injections of Surfagon (a synthetic analog of GnRH, CJSC Mosagrogen, Russia), 9 days apart (on day “0” (50 μg) and on day 9 (50 μg)) and one injection of Magestrofan (500 μg, a synthetic analog of PGF2α CJSC Mosagrogen, Russia) on day 7 of the protocol (Fig. 1).

Additionally, animals in groups 2, 4, and 6 on days “0” and 9 received vitamin complex (Vitamins A, D3, E, and F)-Tetramag 10 ml (CJSC Mosagrogen, Russia) and on day 7-Selevetum (Vitamin E + sodium selenite) in a dose of 10 ml (Belekotekhnika LLC, Belarus), while animals from groups 3 and 5 did not receive any vitamins (Fig. 1).

Similarly, all animals from groups 3–6, starting from the second day of synchronization, were injected subcutaneously with a 5%–20 ml solution of ASD-2f (Armavir Biofactory, Russia), 0.5% novocaine solution (BioFarm Garant LLC, Russia), and saline solution (BIEFFE MEDITAL, S.A., Spain) four times with an interval of 48 h Simultaneously, cows in groups 5 and 6 underwent trans-rectal electrical stimulation of the uterus for 3 min with an electro-ejaculator device (Minitube, Germany) at 7.5 V every second day, while the animals of the first group were left untreated and served as a control (Fig. 1).

### Determination of estradiol-17ß (E2)

Blood samples for determination of E2 concentration were collected from the jugular vein into vacutainers on day 0 and during insemination (day 10). The samples were immediately put on ice and delivered to the laboratory within 3 h. The determination was done at the Veterinary Diagnostic Center (“EQUI LAB” Almaty, Kazakhstan) using an ELISA photometer, the Immunohim-2100 (High Technology, USA), and the XEMA Test System Company Ltd. (Xena Corporate Group, Moscow, Russia).

### Estrus detection and artificial insemination (AI)

The estrus detection during the protocols was done by bulls with aprons. Cows that were allowed to be mounted (standing heat) during the protocol were inseminated the same day. Cows that did not allow mounting until the end of the protocol were AI on day 10 twice at intervals of 10–12 h, The dose of the frozen semen used for AI was 0.25 ml, with a sperm concentration of 15 million/ml. The pregnancy diagnosis was done at day 45 after AI by trans-rectal palpation and confirmed by ultrasound (presence of the embryo). The effectiveness of the synchronization protocols was determined by the estrus manifestation, changes in the genital tract, and a positive pregnancy diagnosis.

### Statistical analaysis

The research data were statistically processed using the RStudio-integrated development environment. The significance for all variables for parametric data was performed with a nonparametric Kruskal-Wallis one-way analysis of variance.

## Results and Discussion

### Estrus manifestation during the synchronization protocols

During the experiment, 17 cows (18.8%) from all groups showed signs of estrus within 5–7 days. The percentage of cows exhibiting signs of estrus in each group was: 33.33% (group 1), 26.6% (group 2), 13.33% (group 3), 6.66% (group 4), 20% (group 5), and 13.33% (group 6, Table 1). Besides standing to be mounted as a sign of estrus, 72.7% of cows in groups 1 and 3, as well as 100% of cows in groups 5 and 6, also found hyperemia of the genital tract, clear vaginal mucus discharge, and uterine rigidity (Table 1).

**Table 1.** Percentage of cows inseminated either at estrus detection or timed (TAI) during the synchronization protocol and pregnancy rates.

Groups	Total number of animals	% of cows AI-ed after spontaneous estrus manifestation			AI results after TAI		
		Total	% of animals in the group	Pregnancy rate (%)	Total	Number of animals	Pregnancy rate (%)
1	15	5	33.3 ± 1.83	60.0 ± 1.10	10	6	60.0 ± 1.55
2	15	4	26.7 ± 1.71	50.0 ± 1.00	11	7	63.6 ± 1.60
3	15	2	13.3 ± 1.32	50.0 ± 0.71	13	8	61.5 ± 1.75
4	15	1	6.7 ± 0.97	100.0	14	9	64.3 ± 1.79
5	15	3	20.0 ± 1.55	100.0	12	9	75.0 ± 1.50
6	15	2	13.3 ± 1.32	100.0	13	10	76.9 ± 1.52

^*^indicated significant differences between the groups (*p* ≤ 0.001)

^**^indicated significant differences between the groups (*p* ≤ 0.001)

**Table 2.** Blood level of estradiol-17β (E2).

Groups	Day 0		Day 10	
	Sample size	nmol/l	Sample size	nmol/l
1	10	1.50 ± 0.20	10	2.19 ± 0.21
2	11	1.47 ± 0.21	11	2.21 ± 0.21
3	13	1.52 ± 0.19	13	2.33 ± 0.18
4	14	1.54 ± 0.20	14	2.44 ± 0.22
5	12	1.55 ± 0.24	12	2.48 ± 0.25
6	13	1.52 ± 0.26	13	2.58 ± 0.17

^*^indicated significant differences between the groups (*p* ≤ 0.05)

^**^indicated significant differences between the groups (*p* ≤ 0.001)

### Detection of the E2 concentration

**Figure 2.**
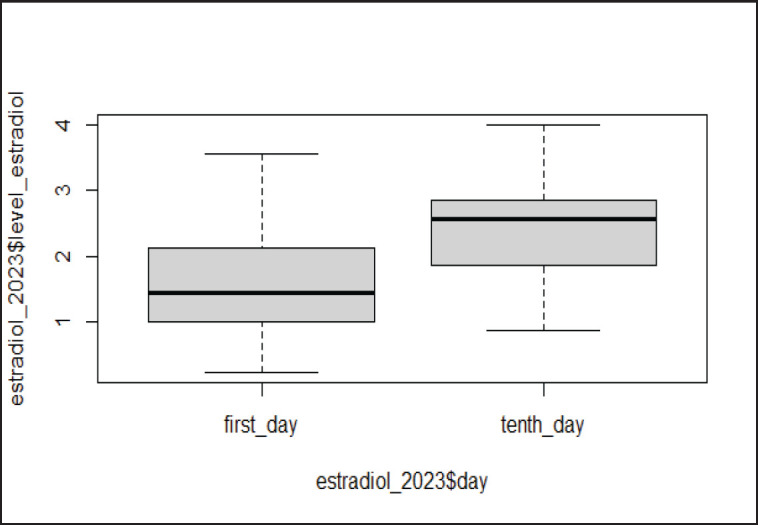
The results of E2 levels. There was a significant difference (*p <* 0.0001***) between E2 levels on day one (1.5) and on day 10 (2.4).

The general distribution for the dependent variable, the level of E2 in the blood of cows, showed that the data were close to normal. The level of E2 in the blood of cows from all groups that completed the protocol on day 0 ranged from 1.47 ± 0.21 to 1.55 ± 0.24 nmol/l (Table 2). On day 0, no differences in the blood level of E2 were observed between the groups. On the 10th day, the blood level of E2 in cows in all groups ranged from 2.19 ± 0.21 to 2.57 ± 0.17 nmol/l. A significant difference was observed in the E2 levels among cows in group 6 compared with groups 2, 3, 4, and the control, while no differences were recorded between groups 5 and 6. When the comparison was done between the same groups on both days (days 0 and 10), a significantly increased blood level of E2 was recorded. It was found that the day factor had a statistically significant influence on the variable E2 level in the blood (Fig. 2). The increase was 46.0% in the control group, 53.3% and 58.4% in groups 3 and 4, as well as 60.0 % and 69.7% in groups 5 and 6, respectively. Furthermore, on the 10th day, there was a 16.7% increase in E2 levels between the control group and the 6th group.

### Pregnancy rate

**Figure 3.**
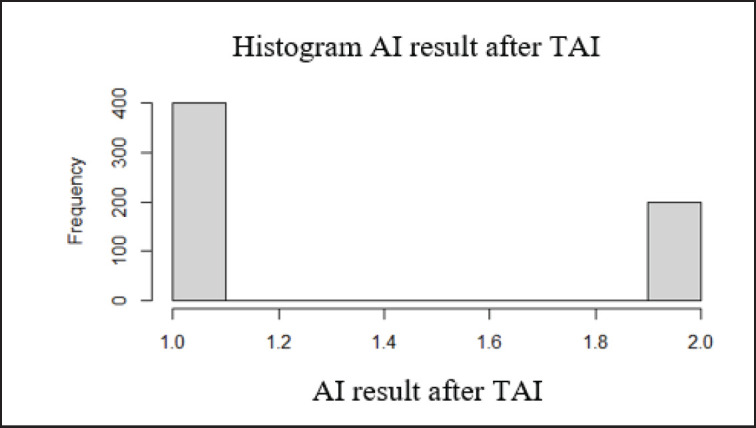
The results of pregnancy after AI. *Note*: on Y-axis pregnancy rate and on X-axis number of cows (%). *p* < 0.05.

**Figure 4.**
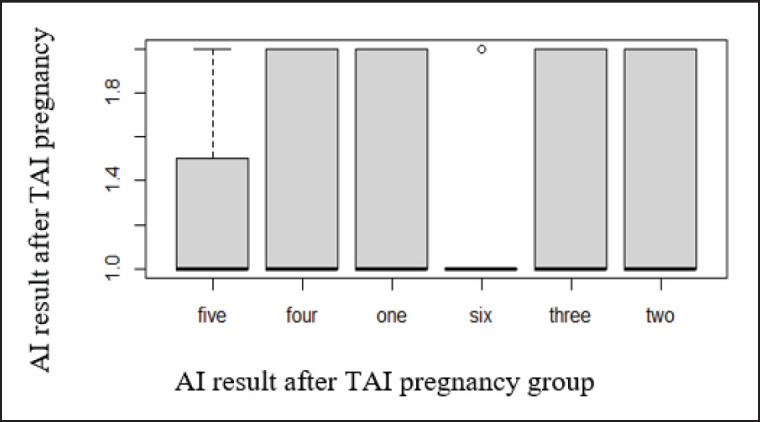
The results of pregnancy after AI. *Note*: on X-axis pregnancy. Y-axis quantity by group.

The pregnancy rates of cows with signs of estrus during the protocol were 50% (groups 2 and 3), 60% (control group), and 100% (groups 5 and 6), respectively (Table 1, Figs. 2 and 3). The pregnancy rates of cows in different groups who completed the protocol were as follows: 60.0% for the control group, 63.6% for the 2nd group, 61.5% for the 3rd group, 64.3% for the 4th group, and 75.0% and 76.9% for the 5th and 6th groups, respectively (Fig. 4). A slight non-significant increase of +3.6%, +1.5%, and 4.3% were observed in groups 2 (addition of Tetramag and Selevetum), group 3 (non-specific tissue stimulator ASD-2f), and group 4 (vitamin complexes and tissue preparation), respectively, compared to the control group. In contrast, a significant increase in the pregnancy rate of 15.0% and 16.9% was observed when electrical stimulation of the reproductive organs was included (groups 5 and 6), respectively, compared to the control group as well as between groups 6 and 4. There was no difference in pregnancy rates between the 5th and 6th groups.

In comparison to the control group, groups (addition of Tetramag and Selevetum), 3 (non-specific tissue stimulator ASD-2f), and 4 (vitamin complexes and tissue preparation) exhibited slight non-significant increases of +3.6%, +1.5%, and 4.3%, respectively, in their pregnancy rates. On the other hand, when electrical stimulation of the reproductive organs was included, a significant increase in pregnancy rates of 15.0% and 16.9% was observed in groups 5 and 6, respectively, compared to the control group and groups 4 and 6. No significant difference in pregnancy rates was found between the 5th and 6th groups.

## Discussion

Increasing the intensification of herd reproduction is one of the main ways to increase the number of beef cattle and beef production and reduce beef costs [23]. The herd’s reproduction’s main objective is to produce one viable calf from one cow annually. The latter could be achieved by the implementation of hormonal methods to control the reproduction function of the animals [24].

There is evidence in the literature that postponing the insemination of cows that have come into heat is fraught with negative consequences for their fertility [25]. Bearing in mind the latter, in our study, all animals that showed spontaneous estrus manifestations during the synchronization protocol were inseminated. The insemination of cows at detected heat in combination with biologically active substances (Tetramag, Selevetum, and ASD-2f) has resulted, on average, in a 65% pregnancy rate. When electrical stimulation of the genitalia was additionally included, the pregnancy rate was 100%. Therefore, it can be noted that electrical stimulation of the genitals in comparison to the use of biologically active substances has a positive effect on the reproductive performance of cows that have spontaneous estrus.

To synchronize the estrous cycle, we have used a combination of Surfagon and Magestrofan, which has resulted in increased synchronization risk. The latter was in agreement with the data in the literature that the combined use of PGF2α and GnRH might increase the synchronization risk and overall fertility of the cows [26,27]. Moreover, the inclusion of biologically active substances (Tetramag, vitamins A, D3, E, and F, and Selevetum, vitamins E and sodium selenite) for estrus synchronization, although inconsistent, had a certain positive effect on estradiol-17ß dynamics and the pregnancy rate of the cows (group 2). Similarly, our results were in contrast with the results reported by Horn et al. [28] that the use of natural and synthetic vitamin E did not affect reproductive efficiency in beef cows. The use of vitamin preparations in the protocol of synchronization of sexual reproduction improved the reproductive functions of animals. At the same time, vitamin preparations activated intrathecal respiration and metabolic processes in the genitalia, improving the function of the endometrium, positively influencing the course of critical periods in the development of the embryo, and thus preventing embryonic mortality. Vitamins promote normal fetal development and the course of pregnancy.

On the contrary, our research supports the report that physical methods of genital stimulation in the form of massage can increase fertility by 5%–7% [29]. Therefore, we used electrical stimulation of the genitals as a physical stimulation method, which positively affected the fertility of the cows. This technique acts at the neuroendocrine and vascular levels. We speculate that during stimulation, the blood flow to the reproductive organs increases, which in turn activates some biochemical processes that lead to increased tone in the reproductive organs as well as stimulate ovulation. In addition, increased blood flow in the reproductive tract stimulated the functional activity of the endometrium, preparing it to receive and promote the sperm, eggs, and subsequently newly formed zygotes. The latter was evidenced by Ganse and Fedotov [30] and Zubova [31], who studied the level of estradiol-17ß during the stimulation, providing an objective picture of the processes occurring in the cow’s body. Interestingly, one study on beef cows demonstrated that clitoral stimulation did not alter pregnancy rates [32]. Biswas et al. [33] found that vulvar and cervical massage and natural mating lead to an increase in intramammary pressure. In the future, we plan to determine the optimal timing of insemination of cows for meat productivity in the conditions of the south-eastern region of Kazakhstan, as well as to continue research on the definition of effective protocols for synchronization.

## Conclusion

The results of our study indicate that electrical stimulation of the genitals with an electro-ejaculator device increases the efficiency of estrus synchronization in beef cows. It can be concluded that the complex use of biologically active substances (Tetramag, Selevetum, and ASD-2f) and electrical stimulation of the genitals using an electro-ejaculator have a positive impact on estrus synchronization and significantly increased efficiency of estrus synchronization that might affect the pregnancy rate in beef cows. However, further experiments involving larger sample sizes are required to provide a more comprehensive understanding of these results.

## List of Abbreviations

ASD-2f, antiseptic-stimulator Dorogov 2 fraction; CL, corpus luteum; GnRH, gonadotropin-releasing hormone; LLC, limited liability company; PGF2α, prostaglandin F2α.
